# Repurposing Antimalarial Pyronaridine as a DNA Repair Inhibitor to Exploit the Full Potential of Gold-Nanoparticle-Mediated Radiation Response

**DOI:** 10.3390/pharmaceutics14122795

**Published:** 2022-12-14

**Authors:** Nolan Jackson, Abdulaziz Alhussan, Kyle Bromma, David Jay, James C. Donnelly, Frederick G. West, Afsaneh Lavasanifar, Michael Weinfeld, Wayne Beckham, Devika B. Chithrani

**Affiliations:** 1 Department of Physics and Astronomy, University of Victoria, Victoria, BC V8P 5C2, Canada; 2 Department of Oncology, Division of Experimental Oncology, Faculty of Medicine & Dentistry, University of Alberta, Edmonton, AB T6G 1Z2, Canada; 3 Department of Chemistry, University of Alberta, Edmonton, AB T6G 2G2, Canada; 4 Faculty of Pharmacy and Pharmaceutical Sciences, University of Alberta, Edmonton, AB T6G 2E1, Canada; 5 British Columbia Cancer-Victoria, Victoria, BC V8R 6V5, Canada; 6 Centre for Advanced Materials and Related Technologies, Department of Chemistry, University of Victoria, Victoria, BC V8P 5C2, Canada; 7 Centre for Biomedical Research, Department of Biology, University of Victoria, Victoria, BC V8P 5C2, Canada; 8 Division of Medical Sciences, University of Victoria, Victoria, BC V8P 5C2, Canada; 9 Department of Computer Science, Mathematics, Physics and Statistics, Okanagan Campus, University of British Columbia, Kelowna, BC V1V 1V7, Canada

**Keywords:** gold nanoparticles, pyronaridine, cancer cells, radiotherapy, DNA repair, ERCC1-XPF, nanomedicine

## Abstract

Radiation therapy (RT) is frequently used to locally treat tumors. One of the major issues in RT is normal tissue toxicity; thus, it is necessary to limit dose escalation for enhanced local control in patients that have locally advanced tumors. Integrating radiosensitizing agents such as gold nanoparticles (GNPs) into RT has been shown to greatly increase the cure rate of solid tumors. The objective of this study was to explore the repurposing of an antimalarial drug, pyronaridine (PYD), as a DNA repair inhibitor to further enhance RT/GNP-induced DNA damage in cancerous cell lines. We were able to achieve inhibitory effects of DNA repair due to PYD at 500 nM concentration. Our results show a significant enhancement in DNA double-strand breaks of 42% in HeLa cells treated with PYD/GNP/RT in comparison to GNP/RT alone when irradiated with a dose of 2 Gy. Furthermore, there was a significant reduction in cellular proliferation for both HeLa and HCT-116 irradiated cells with the combined treatment of PYD/GNP/RT. Therefore, the emergence of promising novel concepts introduced in this study could lay the foundation for the transition of this treatment modality into clinical environments.

## 1. Introduction

Approximately 40% of North Americans will be diagnosed with cancer during their lifetime, and about 1 out of 5 will die from cancer [1,2,3]. Although an increasing number of patients survive at least five years past their cancer diagnosis, cancer continues to be one of the leading causes of death in North America [1,2,3]. One of the main modalities that is used to treat this deadly disease (when not surgically resectable) is RT, with approximately 50% of cancer patients receiving it as a form of treatment [4,5]. RT is an essential element of curative treatment of many cancers including breast, prostate, cervix, head and neck, lung, and brain cancer. However, despite advancements in treatment planning and delivery, we are now approaching the limit of RT dose that can be safely delivered to patients. This creates a clear need for novel methods to enhance tumor control to further improve survival while reducing toxic side effects to patients.

**Figure 1:**
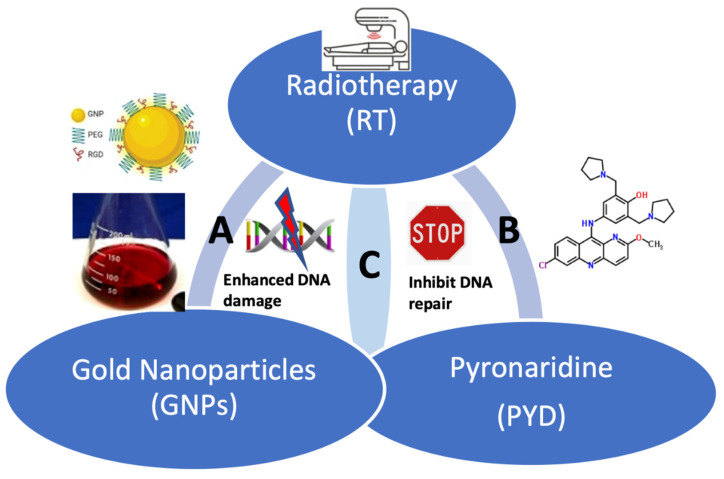
Combination of GNPs, RT, and PYD for a better therapeutic outcome. (**A**) Combination of GNPs with RT. (**B**) Combination of PYD with RT. (**C**) Theme of the current study: a novel combination of GNPs with PYD to further improve the therapeutic outcome in RT.

Enhancing targeted delivery of RT has tremendous potential to maximize the effect of dose given to the tumor and reduce the dose delivered to normal tissue. One of the current strategies to preferentially increase the tumor radiation dose effect is to employ a radiosensitizer to work in unison with RT, which has improved survival for those with cancers [6]. Matsudaira et al. demonstrated that iodine could act as a radiosensitizer of cells in culture over 40 years ago [7]. Furthermore, Santos Mello et al. found that direct injection of iodine into tumors followed by RT suppressed the growth of 80% of tumors [8]. More recently, gold has been pursued as a radiosensitizer due to its higher atomic number (Z) than iodine (Z_Au_ = 79; Z_I_ = 53) and favorable biocompatibility [9]. Irradiation with kilovolt X-rays after intravenous administration of 1.9 nm diameter GNPs to mice bearing subcutaneous tumors was first shown to increase radiocurability of tumors in 2004 by Hainfeld et al. [9]. Since then, there have been many studies establishing the potential of GNPs as a radiosensitizing agent [10,11,12,13,14,15]. GNPs mainly enter cells via endocytosis, and this process depends on the size of the nanoparticle (NP) system, based on both experimental [16] and theoretical studies [17]. It is believed that irradiation of tumors loaded with GNPs results in radiation dose enhancement due to the remarkable increase in the fluence of secondary electrons that are produced from the GNPs. This generates additional free radicals resulting in greater DNA damage, the proximate cause of RT-induced cellular damage [18,19,20,21,22,23,24] as shown in Figure 1A.

In our current study, we incorporated a potential inhibitor of a DNA repair enzyme, ERCC1-XPF, into our current GNP/RT protocol to produce a more efficient GNP-mediated radiosensitization. The ERCC1-XPF complex is a heterodimeric enzyme complex that is a 5′-3′ structure-specific endonuclease involved in several DNA repair pathways in mammalian cells [25]. It is essential for nucleotide excision repair (NER) and has integral roles in interstrand crosslink (ICL) repair as well as double-strand break (DSB) repair. In the ERCC1–XPF heterodimer, ERCC1 regulates the DNA– and protein–protein interactions while XPF provides the endonuclease activity and is involved in DNA binding as well as additional protein–protein interactions [26,27]. Dimerization between the ERCC1 and XPF proteins is essential for endonuclease activity; thus, drugs that can disrupt the dimerization effectively inhibit the enzyme. One of the current therapeutic strategies is to complement RT with the use of DNA repair inhibitors that target the activity, stability, or protein interactions of the ERCC1-XPF enzyme complex as shown in Figure 1B [25,28,29]. By inhibiting this repair enzyme, it is possible to increase the radiotherapy-induced toxicity to cells due to the DNA repair process being hindered.

To take rapid advantage of a potentially efficacious treatment regimen, it is always useful if a clinically approved drug can be repurposed. Due to its structural similarity to previously identified acridine-based inhibitors of ERCC1-XPF, we suspected that the clinically approved anti-malarial drug, pyronaridine (PYD), would be an effective inhibitor of ERCC1-XPF [30,31]. Furthermore, PYD was shown to increase the efficacy of cisplatin when tested in a breast cancer cell line [32]. Although not mentioned by the authors of this report, the mechanism of action of cisplatin is through DNA crosslinking, which also requires ERCC1-XPF for repair [25]. For these reasons, we chose to test PYD as an inhibitor of ERCC1-XPF and examine its efficacy in combination with ionizing radiation, as studies have provided evidence that the heterodimer participates in DNA DSB repair [27,33,34]. Other cell-based studies have been conducted to explore the potential of PYD as an anticancer drug because of its capacity to induce apoptosis and inhibit topoisomerase II [32,35,36]. Our goal is to be able to increase the local effect of radiation dose to the tumor without causing harmful effects on normal tissues. Previous studies have shown that GNPs are ideal candidates to achieve this purpose since they can augment the effect of radiation therapy but induce less toxicity to normal tissues as compared to anticancer drugs such as cisplatin. Furthermore, one of the mechanisms of action of cellular damage due to GNP/RT is via additional DNA damage [37]. Therefore, the addition of PYD to GNP/RT protocol is expected to generate a better therapeutic pathway. The long-term goal is to reduce the dosage of RT and adverse effects associated with this proposed combined treatment to increase the efficacy of RT treatment and improve the quality of life of cancer patients.

In this study, we chose a treatment strategy that can be translated into clinical settings. The GNPs administered to cells were functionalized with polyethylene glycol (PEG) and a peptide that contains the integrin-binding domain RGD. PEG is conjugated to the GNPs through PEGlyation, and it acts as a hydrophobic shield that allows the GNPs to bypass immune system responses by protecting them from aggregation, opsonization, and phagocytosis [38,39]. The addition of RGD facilitates the targeting of tumor cells since many overexpress integrin receptors, thus enabling greater uptake of GNPs into cancer cells, which increases the overall efficacy of the treatment strategy [40,41,42]. Furthermore, clinically feasible nanomolar concentrations of both GNPs and PYD were used throughout this study.

As illustrated in thematic Figure 1C, we incorporated the DNA repair inhibitor, PYD, to further enhance the efficacy of GNP/RT treatment. This study will answer the following questions:

(1) Does PYD inhibit ERCC1-XPF?
(2) Does PYD affect GNP uptake and transportation?
(3) Can we achieve therapeutic benefit using nanomolar concentrations of PYD as opposed to micromolar concentrations?
(4) Is the enhancement of cellular DNA damage of this triple combination, PYD/GNP/RT, significant compared to GNP/RT?

## 2. Materials and Methods

### 2.1. Gold Nanoparticle Synthesis, Functionalization, and Characterization

The GNPs used in this study had a core diameter of ~11 nm and were synthesized using a citrate reduction method. A total of 1.18 mL of 1% tetra chloroauric acid (HAuCl_4_) was added to an Erlenmeyer flask containing 28.82 mL of ddH_2_O while being heated on a hot plate and stirred vigorously, creating a yellow solution. Once the solution came to a boil, 1.2 mL of 5% sodium citrate tribasic dihydrate (HOC(COONa)(CH_2_COONa)_2_·2H_2_O) was quickly added to the flask. After the color of the solution became ruby red, indicating the synthesis of GNPs, it continued to boil and be stirred for another 5 min. The GNP solution was then brought to room temperature while being stirred.

Polyethylene glycol (PEG) and peptide containing RGD (CKKKKKKGGRGDMFG) integrin-binding domain, with sizes of 2000 Da and 1600 Da, respectively, were used to functionalize the GNPs. PEG was added such that the grafting density was 1 PEG per nm^2^ of the GNP surface area. It was added into the GNP solution and then stirred. After PEGylation, RGD was added to the GNP + PEG solution at a ratio of one molecule of RGD for every two molecules of PEG to create the GNP+PEG/RGD complex. The RGD peptide has a cysteine at the end, and therefore it binds to GNP surface via a gold-thiol bond [43].

Characterization was performed using ultraviolet-visible (UV-VIS) spectrometry (Perkin Elmer λ 365 Spectrophotometer, Waltham, MA, USA) to estimate the size and concentration of the GNPs. The hydrodynamic radius and surface charge of the GNP, GNP + PEG, and GNP + PEG/RGD complexes were analyzed using dynamic light scattering (DLS) and ζ potential (Anton Paar LiteSizer 500, Graz, Austria), respectively. Verification of the shape and size was performed using Transmission Electron Microscopy (TEM) (Ultra-high Resolution Scanning Electron Microscope SU9000 Ultra-high Resolution Scanning Electron Microscope, Hitachi, Pleasanton, CA, USA).

### 2.2. ERCC1-XPF Incision Assay

The purification of recombinant human ERCC1-XPF protein and the fluorescence-based incision assay for ERCC1-XPF enzymatic activity were carried out as previously described [29]. Steady-state fluorescence used to obtain the *K_d_* value for PYD affinity for ERCC1-XPF was performed as previously described [29,30].

### 2.3. Cell and Culture Conditions

Human colorectal carcinoma cell line HCT-116 (ATCC#: CCL-247™) and cervical cancer cell line HeLa (ATCC#: CCL-2™) were purchased from the American Type Culture Centre (ATCC). Both cell lines were cultured in high-glucose Dulbecco’s modified Eagle medium (DMEM; Gibco, 11965092; Gibco, ThermoFisher Scientific, Waltham, MA, USA)) augmented with 10% Fetal Bovine Serum (Gibco), 4 mM GlutaMax (Gibco), and 1% penicillin/streptomycin (Gibco). For cell washing and cell detachment, phosphate-buffered saline (PBS) and TrpyLE (Gibco) were used, respectively. All cells were incubated at 37 °C with 5% CO_2_.

### 2.4. Proximity Ligation Assay

Disruption of ERCC1-XPF heterodimerization in HCT-116 cells was examined by the proximity ligation assay as previously described [29,44].

### 2.5. Live Cell Imaging

Cells were plated in 3 cm coverslip-bottom dishes for both cell lines. Cells were dosed with either Cy5-labeled 5 (excitation 633 nm, emission filter 650 nm LP) GNP complex, 100 nM PYD/GNP complex, or 500 nM PYD/GNP complex and incubated for 24 h. Twenty minutes prior to imaging, dishes were stained with NucBlue™ Live ReadyProbes™ Reagent ((R37605; ThermoFisher Scientific, Waltham, MA, USA)) containing Hoechst 33,342 dye. Images were taken with a confocal laser scanning microscope (Zeiss LSM 980, Carl Zeiss Microscopy GmbH, Jena, Germany) using a 60× oil immersion objective lens.

### 2.6. DNA DSB Assay/Dark-Field Imaging and HIS

By using fluorescently labeled antibodies against the repair proteins 53BP1 and γ-H2AX, the DNA DSB damage was able to be observed. Using a solution of 0.5% BSA/0.1% Triton-X/PBS, primary antibody and secondary antibody diluted solutions were made. Primary antibody solutions were diluted 1:200, whereas secondary antibody solutions were diluted 1:500. Cells were plated on glass coverslips in 6-well dishes and incubated for 24 h to adhere. Cells were then dosed with GNP complex and PYD at concentrations of 7.5 µg/mL and 500 nM, respectively, and incubated. One well was left un-dosed for the control sample. Twenty-four hours after treatment, cells were washed twice with PBS and then fixed with 4% PFA. Following fixation, the cells were treated with 2% BSA/0.1% Triton-X in PBS for 20 min for blocking. Primary antibody diluted solution was added onto parafilm where the coverslips were removed from the dishes and placed face down onto the solution and were then incubated in the dark at room temperature for 1 h. Slips were returned to the wells, and cells were washed with PBS twice, followed by another wash with 0.5% BSA/0.1% Triton-X/PBS. Secondary diluted antibody solutions were then added onto parafilm where the coverslips were placed face down onto the solutions and incubated for 30 min in the dark at room temperature. Post incubation, cells were washed twice with PBS, and then glass coverslips were mounted with ProLong™ (P36930; ThermoFisher Scientific, Waltham, MA, USA) Glass Antifade Mountant for imaging. Imaging for the DNA DSB assay was performed with a confocal laser scanning microscope (Zeiss LSM 980) using a 60× oil immersion objective lens. Dark-field images were taken with a 60× oil immersion objective lens, and for HSI, a hyperspectral camera (CytoViva, Auburn, AL, USA) was used.

### 2.7. Cellular Uptake of Gold Nanoparticles

For both cell lines, cells were plated in 6-well dishes and incubated for 24 h. Cells were then treated with either GNP complex, 100 nM PYD/GNP complex, or 500 nM PYD/GNP complex, and the samples were incubated for 24 h. Cells were dosed with GNPs at a concentration of 7.5 µg/mL. For each condition, 3 wells were used. Twenty-four hours post dosing, cells were washed three times with PBS before being dissociated with TrypLE (Gibco) and fresh media being added. A total of 1 mL of each sample was transferred into 1.5 mL Eppendorf tubes. The cell count per mL was then carefully counted for each sample using a hemocytometer. Samples were stored at 4 °C until further cell processing.

A total of 400 uL of each sample was added to glass tubes for processing. Aqua regia (3:1 molar ratio of HCl and HNO3 (VWR)) was added to each sample before being placed into a heated mineral oil bath for ~30 min at 90 °C. Hydrogen peroxide was then added to further ensure digestion of both cells and GNPs. Lastly, each sample was diluted with deionized water to a 2.5% *v*/*v* (volume per volume) acid content, and the gold content in each sample was measured using inductively coupled plasma–mass spectrometry (ICP-MS; Agilent 8800 Triple Quadrupole, Agilent Technologies, Santa Clara, CA, USA). The number of gold nanoparticles per cell is calculated by the following:

$$\frac{\frac{Gold\;Conc\mathrm{entration}}{Sample}\left[\frac{g}{\mathrm{mL}}\right]\times Sample\;Vol\mathrm{ume}\;\left[\mathrm{mL}\right]\times N_{A}\left[\frac{\mathrm{atoms}}{\mathrm{mol}}\right]}{Gold\;atomic\;mass\left[\frac{g}{\mathrm{mol}}\right]\times \mathrm{Number}\;of\;Cells\times \frac{\;Gold\;atoms}{Gold\;nanoparticle}}$$

where *N_A_* is Avogadro’s number. The number of gold atoms per GNP is calculated by the following:

$$\frac{Atoms\;per\;unit\;cell\times Gold\;Nanoparticle\;Vol\mathrm{ume}\;\left[{\mathrm{nm}}^{3}\right]}{Unit\;cell\;Vol\mathrm{ume}\;\left[{\mathrm{nm}}^{3}\right]}=\frac{4\times \frac{4\pi r^{3}}{3}}{a^{3}}=\frac{2}{3}\pi {\left(\frac{D}{a}\right)}^{3}$$

where *a* = 0.408 nm, the length of a unit cell, and *D* is the core diameter of the spherical GNPs, measured to be 10.8 nm. The GNPs were synthesized using a citrate reduction method that results in a face-centered cubic lattice with 4 atoms per unit cell. For the calculations, it is assumed that the size of the GNPs is homogenous.

### 2.8. Proliferation Assay

For each cell line, cells were plated in 96-well plates. For each irradiation treatment condition, 0 Gy and 2 Gy, cells were dosed with either GNP complex, 500 nM of PYD, or 500 nM PYD/GNP complex. Cells were dosed with GNPs at a concentration of GNPs 7.5 µg/mL. Wells were also left un-dosed for the control sample. Twenty-four hours after dosing, the cells were irradiated with the corresponding treatment condition. Following irradiation, media were removed from each well, and fresh media were added. Cell viability was measured with a CytationOne™ Multi-Reader (filters at excitation of 530/25 nm and emission of 590/35 nm, Winooski, VT, USA). Prior to measurements, media were removed from wells that were to be measured, and a solution of 10% PrestoBlue™ (A13261; Thermo-Fisher, Waltham, MA, USA) in media was added, and cells were incubated for 2 h at 37 °C.

### 2.9. Cellular Irradiation

All irradiation of samples was performed using a clinical 6 MV linear accelerator (Varian Truebeam, Palo Alto, CA, USA). All samples were placed between two 30 cm × 30 cm × 5 cm solid water blocks and irradiated with a dose of 2 Gy. Non-irradiated samples were also brought to the location of the linear accelerator to maintain consistency.

### 2.10. Statistical Analysis

Statistical analysis was performed using the Mann–Whitney test via the python package statannot (O.2.3). For statistical significance represented in figures, ns indicates no significance, * indicates 0.01 < *p* < 0.05, ** indicates 0.001 < *p* < 0.01, and *** indicates *p* < 0.001. All experiments were repeated three times and the data presented are the average of all experiments. Error bars signify one standard deviation from the mean of the three independent measurements.

## 3. Results and Discussion

### 3.1. Characterization of GNPs

**Figure 2:**
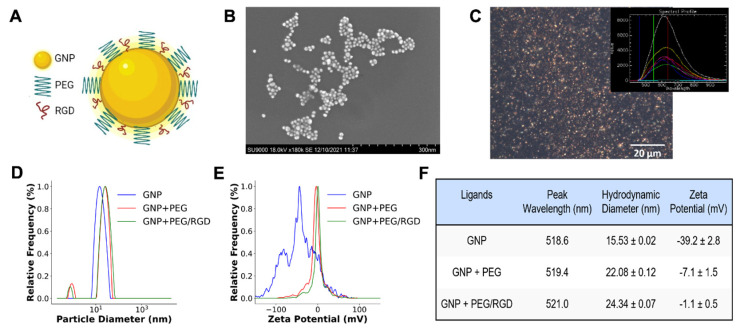
Characterization of GNPs. (**A**) Schematic diagram of GNPs functionalized with PEG and RGD peptide. (**B**) Transmission electron microscopy (TEM) image of GNPs. (**C**) Dark-field image of GNPs with inset figure displaying spectra collected using hyperspectral imaging. (**D**,**E**) Dynamic light scattering and zeta potential data for GNPs at each step of the functionalization process. (**F**) Summary of characterization data.

GNPs were synthesized using a citrate reduction method and functionalized with both polyethylene glycol (PEG) and a peptide containing integrin domain, RGD (RGD peptide), for biocompatibility and targeting, respectively (see schematic Figure 2A) [45,46,47]. The addition of PEG increases the circulation time in the body by preventing macrophage uptake via a hydrophobic shield [39]. It also provides stability for conjugation with the integrin-binding domain RGD that is used for targeting purposes, which increases the efficacy of the treatment modality since many tumor cells overexpress integrins [40]. GNPs with a core diameter of ~11 nm were used for this study as data have shown that smaller NPs are more efficient in penetrating tissues than larger NPs [48,49]. GNPs were first functionalized with PEG followed by RGD peptides to create the GNP + PEG/RGD complex. This functionalizing process was chosen for the GNPs since it is applicable for future in vivo and clinical trials based on preclinical data [45,50].

Transmission electron microscopy (TEM) images were taken to verify the size of GNPs as shown in Figure 2B. GNPs are also used as very effective contrast agents for hyperspectral imaging due to their highly reflective nature of visible light. Figure 2C displays a hyperspectral image of GNPs from a dark-field microscope as well as spectra collected from them as shown in the inset figure. This technique is useful as it allows one to map the localization of GNPs in cells, which was used to verify the intracellular distribution of GNPs in cells as discussed below. To characterize the GNPs, measurements using ultraviolet-visible spectrometry (UV-VIS), dynamic light scattering (DLS), and zeta potential were made at each step of the functionalization process (Figure 2D–F). The estimation of size and concentration of the GNPs was performed using UV-VIS. The citrate-capped GNPs were measured to have a peak wavelength of 518.6 nm, suggesting a core diameter of ~11 nm [51,52]. After functionalization with PEG and RGD peptides, the peaks of UV spectra slightly redshifted (Table in Figure 2F and Supplementary Section S1). The hydrodynamic diameter of the standard GNPs was 15.53 ± 0.02 nm as measured by DLS (Figure 2D). The measured hydrodynamic diameter increased to 22.08 ± 0.12 nm after PEGylation and further increased to 24.34 ± 0.07 following the conjugation with RGD. The addition of PEG and RGD also significantly reduced the overall negative charge of the citrate-stabilized GNPs as the negative citrate molecules were replaced by neutral PEG molecules and positive RGD molecules (Figure 2E). Measurements of the zeta potential confirmed this as the surface charge of −39.2 ± 2.8 mV for the citrate GNPs was reduced to −7.1 ± 1.5 mV and −1.1 ± 0.5 mV with the addition of PEG and RGD, respectively, further verifying successful conjugation. The table in Figure 2F summarizes the characterization data corresponding to the GNP functionalization process. It was also found that the GNP complex did not have any significant interaction with PYD (Supplementary Section S2).

### 3.2. PYD Is an Inhibitor of ERCC1-XPF

**Figure 3:**
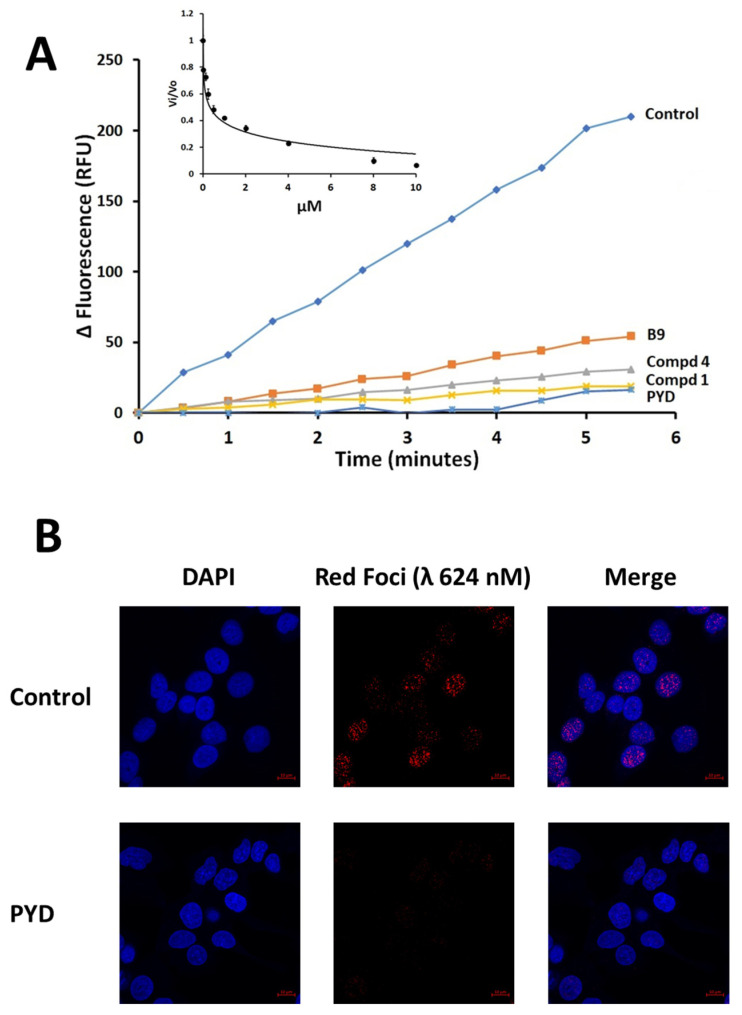
Inhibition of ERCC1-XPF by pyronaridine (PYD). (**A**) Representative plots of fluorescence generated by ERCC1-XPF catalyzed incision of a fluorescently quenched stem-loop DNA substrate containing fluorescein (FAM) and dabcyl groups at the 5′- and 3′-termini, respectively. The incision releases a FAM-labeled oligonucleotide from the quenching caused by close juxtaposition to the dabcyl group. Control represents the substrate incision by the uninhibited enzyme exposed only to the equivalent amount of DMSO vehicle. Other compounds shown for comparison are B9: 2-chloro-9-((3-((4-(2-(dimethylamino)ethyl)piperazin-1-yl)methyl)-4-hydroxyphenyl)amino)acridin-2-ol; Cmpd4: 4-((6-chloro-2-methoxyacridin-9-yl) amino)-2-((4-(2-(dimethylamino)ethyl) piperazin-1-yl) methyl)phenol; Cmpd1: 4-((6-chloro-2-methoxyacridin-9-yl)amino)-2-((4-(2-(diisopropylamino)ethyl)piperazin-1-yl)methyl)phenol. The inset shows the initial velocities (slopes) normalized by its value in the absence of compound vs. its value in the presence of increasing µM concentrations of PYD. The bars represent the S.D. of three different measurements for each point (R^2^ = 0.95). (**B**) Representative PLA images of HCT-116 cells exposed to 2 μM of pyronaridine (PYD) or the equivalent amount of DMSO vehicle (1 μL/mL, Control). Images were obtained at 40× magnification. ERCC1-XPF complexes appear as red dots, and cellular nuclei are shown in blue after DAPI staining.

To assess the inhibitory capacity of PYD against ERCC1-XPF, we employed a fluorescence-based assay, in which the recombinant enzyme cleaves a stem-loop structure to release, and thereby unquench, a 5′-FAM-tagged oligonucleotide [53,54]. As shown in Figure 3A, PYD markedly slowed the rate of release of the fluorescent oligonucleotide compared to the uninhibited control. Also included in Figure 3A are data previously obtained with three acridine-based inhibitors, i.e., B9, compound 4, and compound 1, indicating that inhibition by PYD compares favorably to inhibition by these compounds [29,30,55]. From the inset plot, an IC_50_ of 0.321 ± 0.022 µM was calculated for PYD. A *K_d_* value of 150 ± 10 nM for the binding of PYD to ERCC1-XPF was obtained by steady-state fluorescence (data not shown). We used the proximity ligation assay to determine if PYD disrupts the ERCC1-XPF heterodimer in HCT-116 cells. As is evident from Figure 3B, PYD induced a sharp reduction in the number of fluorescent foci arising from dimerized ERCC1-XPF in comparison to the signal observed with the control vehicle-treated cells.

### 3.3. Cellular Uptake of GNPs in the Presence of PYD

**Figure 4:**
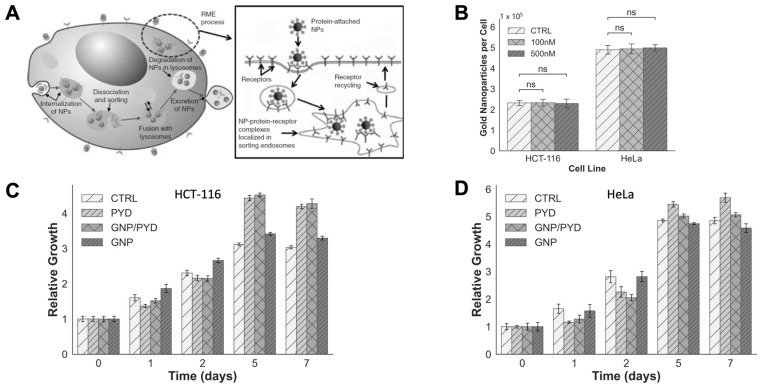
Cellular uptake and transport of GNPs. (**A**) Schematic diagram illustrating the endo-lyso path of GNPs within a cell, and inset figure further illustrates the endocytosis process. (**B**) Comparison of the cellular uptake of GNPs in the presence and absence of PYD. (**C**,**D**) Relative growth in the presence of GNPs and 500 nM of PYD for HCT-116 and HeLa, respectively. Cells were dosed with GNPs at concentration of 7.5 µg/mL. “ns” indicates no significance.

One of the major pathways for cellular uptake of GNPs is via endocytosis where GNPs are engulfed by the cellular membrane through membrane invaginations [56,57,58]. In particular, the main modality in which GNPs experience cellular uptake is through receptor-mediated endocytosis (RME) as illustrated in Figure 4A [57,59]. After the endocytosis process, GNPs get trapped in endosomes, which is followed by the fusing with lysosomes for further processing before their excretion from the cell through the exocytosis process. One integral component in RME is the cellular membrane wrapping of GNPs as it requires the curvature of the membrane to be warped, increasing the elastic energy of the membrane [58,59]. Another driving force in RME is the recruitment of surface receptors to binding sites. As the free energy decreases due to immobilization of receptors from ligand-receptor binding, receptors can diffuse to wrapping sites to aid in the completion of the membrane wrapping [60]. This results in the cellular uptake being a direct result of the competition between the free energy required to drive GNPs into cells and the recruitment of receptors to binding sites [58]. It has been shown that GNPs with a diameter of 50 nm have the highest uptake in the size range of 10–100 nm [16]. With the introduction of PEG and RGD peptides onto the surface of GNPs, smaller GNPs have better uptake than 50 nm GNPs [49,61,62,63,64]. Higher curvature of smaller NPs allowed RGD peptide to access the targeted surface receptors despite PEG (2000 Da) being slightly larger than RGD peptide (1600 Da). However, this size difference in PEG and RGD played a significant role in reducing the targeting ability of 50 nm GNPs. This is due to the lower curvature of NP surface allowing PEG to screen RGD peptide as discussed in our previous work [49,61,62,63]. We also found that these smaller NPs penetrated better tumor tissue making them more durable for in vivo studies [45,46,49]. We were able to successfully test these smaller GNPs functionalized with PEG and RGD in our in vivo studies [64,65]. Therefore, we chose smaller GNPs of ~11 nm in diameter since our next goal is to test this treatment strategy, as described in our study, using three-dimensional tissue models followed by animal models.

As we have shown, PYD could be pharmacologically targeted against cancer as it has the capacity to inhibit ERCC1-XPF. The goal of this study is to evaluate the potential of repurposing this antimalarial drug, PYD, into an anticancer drug to exploit the full potential of GNP-mediated RT. We chose a cervical cancer cell line, HeLa, and colorectal cancer cell line, HCT-116, as our tumor models for this study. A key component in GNP-enhanced RT is to ensure the cellular uptake of GNPs; thus, we tested whether the addition of PYD influences the GNP uptake and transport within treated cells. To quantify the cellular uptake of GNPs, cells were dosed with a concentration of 7.5 µg/mL of GNPs, and inductively coupled plasma spectroscopy (ICP-MS) was used for the analysis of the uptake. Based on our quantification data in Figure 4B, PYD did not affect the uptake of GNPs significantly at both 100 and 500 nM concentrations as expected. However, the data showed that there was a significant difference in the cellular uptake of GNPs between the cell lines. This could potentially be attributed to the difference in size of the cells and the possible variations in the integrins that are expressed between the cell lines [66]. The intracellular distribution of GNPs also raises concerns about potential cytotoxicity that they may induce. Therefore, we chose a concentration of GNPs used in this study that is clinically feasible [67,68]. However, with the combination of both PYD and GNPs, it is unknown whether this would induce further toxicity to cells. To verify the effect of PYD/GNPs within cells, we monitored the cell proliferation as illustrated in Figure 4C,D for both HCT-116 and HeLa at 500 nM concentrations of PYD. Our results show that cells treated with PYD were not susceptible to an increase in any potential toxicity that this treatment technique could induce to cells due to the presence of GNPs. Since radiation was not administered to the cells, there was no definitive trend between the relative growth of cells treated with GNP/PYD versus solely PYD as the GNPs’ role is to act as a radiosensitizer.

**Figure 5:**
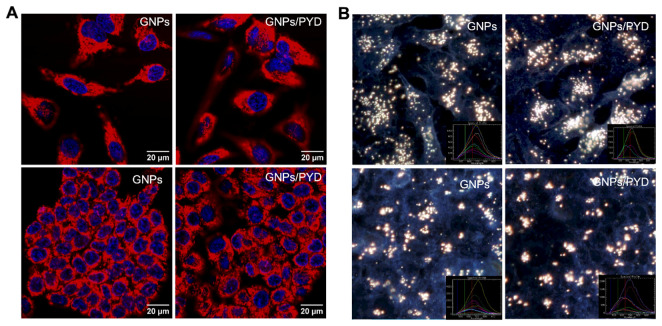
Qualitative analysis of GNP uptake in the absence and presence of PYD in HeLa (top) and HCT-116 (bottom) cells. (**A**) Live cell confocal images of intracellular distribution of GNPs in HeLa and HCT-116. The left and right columns show the distribution of GNPs in the absence and presence of PYD, respectively. Red and blue colors represent GNPs and nuclei stain, respectively. (**B**) Hyperspectral images of intracellular distribution of GNPs in HeLa and HCT-116. The left and right columns show the distribution of GNPs in the absence and presence of PYD, respectively. The inset figure in each image shows the spectra collected from GNP clusters. Cells were dosed with GNPs and PYD at a concentration of 7.5 µg/mL and 500 nM, respectively.

To further support quantification data in Figure 4B, we used confocal and hyperspectral imaging for visualizing intracellular distribution of GNPs (see Figure 5). There were no significant differences in GNP uptake and distribution in the presence of PYD based on confocal and hyperspectral images in Figure 5. Therefore, we can conclude that PYD does not affect intracellular GNP uptake and transport. It is also worth noting that the images do display discrepancies between the uptake of gold between the different cell lines supporting the significant difference between the amount of gold observed in our quantification data. The results we obtained indicate that PYD does not affect the proliferation of gold-bearing cells or the GNP uptake and its intracellular distribution at the concentrations used in this study. This is very promising since this is the first time that free PYD has been tested with GNPs as a combined treatment. PYD is already currently being used with other anticancer drugs to overcome multidrug resistance. A previous study has used gold nanorods for simultaneous delivery of PYD and doxorubicin to overcome multidrug drug resistance in breast cancer. A near-infrared (NIR) laser was used for the release of the drugs from gold nanorods [69]. However, in our study, we used spherical GNPs with free PYD to enhance the GNP-mediated radiosensitization at clinically relevant MeV energies as discussed next. We believe this modality can be translated at a much quicker pace to clinical work since linear accelerator set-ups used for our radiation study are already used in the clinic.

### 3.4. Evaluation of the Triple Combination of RT, GNPs, and Pyronaridine

**Figure 6:**
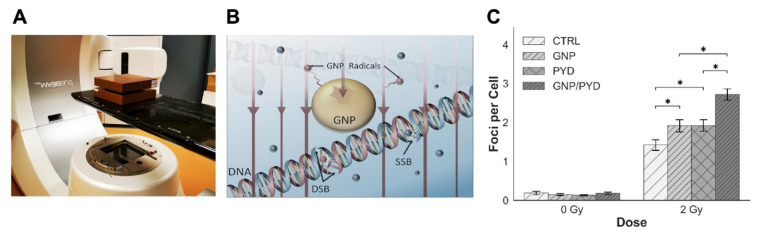
Evaluation of the triple combination of RT/GNP/PYD. (**A**) Linear accelerator set-up used for the radiation treatment of samples. (**B**) Schematic diagram highlighting the enhancement of DNA damage due to the increased presence of radicals (marked in red) in the presence of GNPs during RT. (**C**) Quantitative assessment of DNA DSBs 24 h post irradiation with dose of 2 Gy using 53BP1 as a marker in HeLa cells. Cells were dosed with GNPs and PYD at a concentration of 7.5 µg/mL and 500 nM, respectively. * indicates 0.01 < *p* < 0.05.

Studies have shown that GNPs can be used as a radiation dose enhancer with MeV photon beams due to an increasing beam softening with depth in tissue [70]. In our study, we incorporated PYD and GNPs into RT to further exploit the potential benefits of RT. A 6 MV clinical linear accelerator (see the set-up in Figure 6A) was used to deliver a more clinically relevant single-dose fraction of 2 Gy. For clinical treatments, RT regimens consist of ~20 to 30 fractions of 2 Gy, so a single fraction was used for our study to simulate a clinically relevant treatment environment. It has been previously shown that GNPs localized intracellularly have the aptitude to increase the probability of ionization events leading to locally enhanced depositions of energy that increases the level of RT-induced DNA damage as illustrated in Figure 6B [71]. The main modality in which this is accomplished is that the presence of GNPs creates a shower of secondary electrons that interact with water molecules to further increase the production of DNA-damaging species such as free radicals [71,72,73]. By incorporating GNPs into tumor cells, it is possible to locally increase the damage delivered to them since the secondary electrons can only travel a few micrometers. This is essential as we are currently approaching the limit of dose that can be safely administered to patients; thus, a novel approach that can locally increase dose is necessary to overcome this hurdle. It has been shown that a radiation dose of 2 Gy with a 6 MV photon beam resulted in a further 30% decrease in survival vs. irradiated controls when GNPs were incorporated within cells [74,75]. Therefore, we proposed that the incorporation of a DNA repair inhibitor such as PYD could make a substantial contribution to the efficacy of RT and provide a breakthrough in GNP/RT treatment approach.

**Figure 7:**
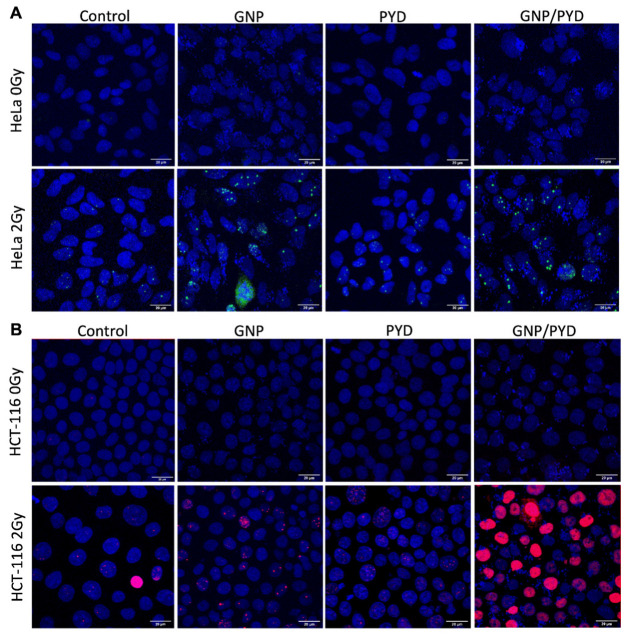
Mapping of DNA DSBs 24 h post irradiation to evaluate the triple combination of RT/GNP/PYD. (**A**,**B**) Qualitative assessment of DNA DSBs for HeLa and HCT-116, respectively. Cells were irradiated at 6 MV with a dose of 2 Gy and treated with GNPs and PYD at a concentration of 7.5 µg/mL and 500 nM, respectively. Scale bar = 20 µm. 53BP1 and γH2AX foci appear as green and red dots, respectively and cellular nuclei are shown in blue after DAPI staining.

In our study, cell cultures were dosed with a clinically feasible concentration of 7.5 µg/mL of GNPs and 500 nM of PYD 24 h prior to radiation. To illuminate the efficacy of the treatment, we mapped the DNA DSBs to highlight the effect of the triple combination of GNPs/RT/PYD. By mapping two DNA repair proteins, 53BP1 and γH2AX, the DNA DSB damage can be assessed. In the case of Hela cells, we were able to visualize both 53BP1 and γH2AX foci (see Supplement Figure S3). However, only the 53BP1 marker was used for quantification of foci in HeLa cells since previous studies have shown that γH2AX foci are not always associated with DNA DSBs [76,77]. According to our results illustrated in Figure 6C and Figure 7A, there was a significant enhancement of DNA DSBs of 42% in HeLa cells treated with the strategy of GNP/PYD/RT in comparison to the GNP/RT protocol. In the case of HCT-116 cells, we were able to map only γH2AX, but we were unable to quantify the DNA DSBs due to the saturation of foci we observed with the triple combination of GNP/PYD/RT, but, as illustrated in Figure 7B, a significant increase in DNA DSBs can be seen with the triple combination of GNP/PYD/RT as compared to both GNP/RT and PYD/RT. Thus, the results found display an increase in DNA damage in both cell lines with the addition of PYD to GNP-mediated RT. It is known that one of the mechanisms of cell damage due to GNPs with radiation is via DNA DSBs [11]. Therefore, this significant increase in DNA DSBs in the presence of 500 nM concentrations of PYD indicates that PYD is a potent inhibitor of a DNA repair process [27,28,30,44]. Recent interest in DNA repair enzymes has been fueled by the success of inhibitors of poly(ADP-ribose) polymerase (PARP), an enzyme involved in DNA single- and double-strand break repair, in the treatment of many solid tumors. These results suggest that PYD could be used as a viable option for DNA repair inhibition for future clinical applications.

**Figure 8:**
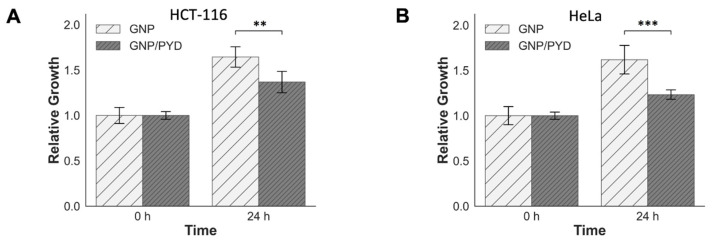
Relative growth of cells after a 2 Gy radiation dose at 6 MV delivered by a clinical linear accelerator. (**A**,**B**) Reduction in relative growth 24 h post irradiation for HeLa and HCT-116 cells, respectively. Cells were dosed with GNPs and PYD at a concentration of 7.5 µg/mL and 500 nM, respectively. ** indicates 0.001 < *p* < 0.01, *** indicates *p* < 0.001.

To further analyze the efficacy of this treatment modality, the relative growth differences 24 h post irradiation were measured considering that a dose of 2 Gy is given daily in a fractionated dose treatment plan (see Figure 8). Consistent with the DNA DSB quantification data, a significant reduction was observed in the relative growth for HCT-116 and HeLa cells treated with GNP/PYD/RT 24 h after the treatment, clearly emulating an increase in the induced radiotherapeutic damage delivered to cells.

It is clear from our results that PYD could offer significant therapeutic benefits to both RT and GNP-enhanced RT. This novel approach could pave the way for better outcomes in RT as PYD is already a clinically approved drug, and the benefits can be achieved at a lower concentration of the drug (nanomolar vs. micromolar), and by complementing the treatment with GNPs, it is possible to target tumors and locally increase the dose deposited to them, establishing this treatment modality as a feasible option for future clinical applications.

## 4. Conclusions

Radiotherapy (RT) is one of the most widely applied treatment approaches, being used for approximately 50% of all cancer patients worldwide. GNP targeting of tumor cells is shown to be effective in vitro and in clinical trials by selectively increasing the RT deposited dose, thus increasing the therapeutic index. Pyronaridine is an antimalarial drug that is clinically approved that can be repurposed for cancer treatment. In this study, we observed that repurposing of PYD, at nanomolar concentrations, as a DNA repair inhibitor shows promise to further improve GNP-enhanced radiosensitization. Therefore, a novel combined modality of PYD/GNP/RT can lead to limited or non-existent side effects, hence, improving the quality of life of cancer patients. Our future goal is to test this novel technology using three-dimensional in vitro models followed by relevant in vivo models to accelerate its use in future clinical trials.

## Data Availability

Datasets generated and/or analyzed during the current study are available from the corresponding author on reasonable request.

## Supplementary Materials

The following supporting information can be downloaded at: https://www.mdpi.com/article/10.3390/pharmaceutics14122795/s1, Figure S1: UV Visible spectra for GNP, GNP-PEG and GNP-PEG-RGD; Figure S2: (A–C) UV Visible spectra, Dynamic light scattering and zeta potential data, respectively, for GNP complex and GNP complex in the presence of PYD; Figure S3: 53BP1 and γH2AX foci for HeLa cells treated with a 2 Gy radiation dose; Figure S4: Relative growth of cells after a 2 Gy radiation dose at 6 MV delivered by a clinical linear accelerator. (A,B) Reduction in relative growth 24 h post irradiation for HeLa and HCT-116 cells, respectively. Cells were dosed with PYD at a concentration of 500 nM.
