# Supplementary material for: Repurposing Antimalarial Pyronaridine as a DNA Repair Inhibitor to Exploit the Full Potential of Gold-Nanoparticle-Mediated Radiation Response
Source: Pharmaceutics. 2022 Dec 14;14(12):2795. doi: 10.3390/pharmaceutics14122795 (PMC9783290; doi:10.3390/pharmaceutics14122795)
Supplement: Supplementary file 1 [file pharmaceutics-14-02795-s001.zip › pharmaceutics-2003624-supplementary.pdf]

Repurposing Antimalarial Pyronaridine as a DNA Repair Inhibitor to Exploit the Full Potential of Gold-Nanoparticle-Mediated Radiation Response

Nolan Jackson [1], Abdulaziz Alhussan [1], Kyle Bromma [1], David Jay [2], James C. Donnelly [3], Frederick G. West [2,3], Afsaneh Lavasanifar [4], Michael Weinfeld [2], Wayne Beckham [1,5] and Devika B. Chithrani [1,6,7,8,9,*]

Supplementary section S1: UV Visible spectroscopy data

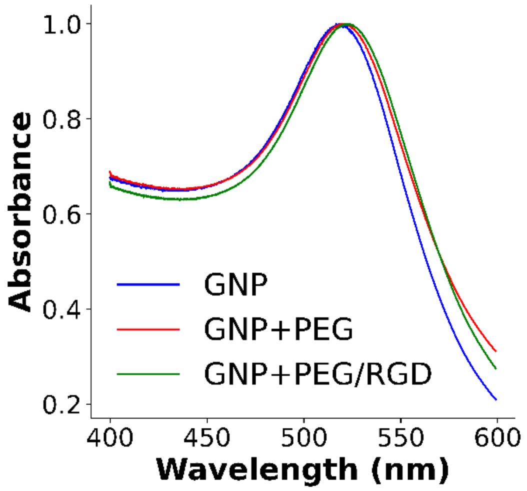

Figure S1. UV Visible spectra for GNP, GNP-PEG and GNP-PEG-RGD.

Supplementary section S2: Characterization of GNP complex in presence of PYD

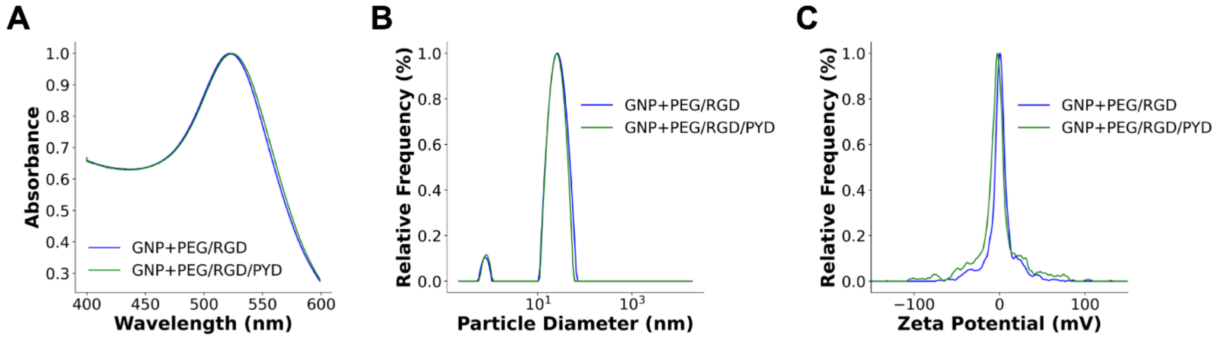

Figure S2. A-C) UV Visible spectra, Dynamic light scattering and zeta potential data, respectively, for GNP complex and GNP complex in the presence of PYD.

Supplementary section S3: Co-localization of foci

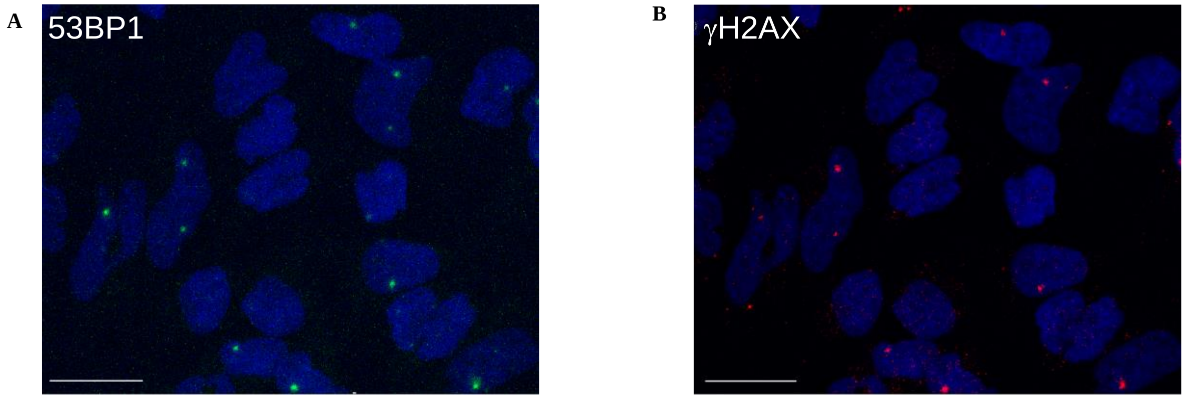

Figure S3. 53BP1 and γH2AX foci for HeLa cells treated with a 2 Gy radiation dose.

Supplementary section S4: Characterization of GNP complex in presence of PYD

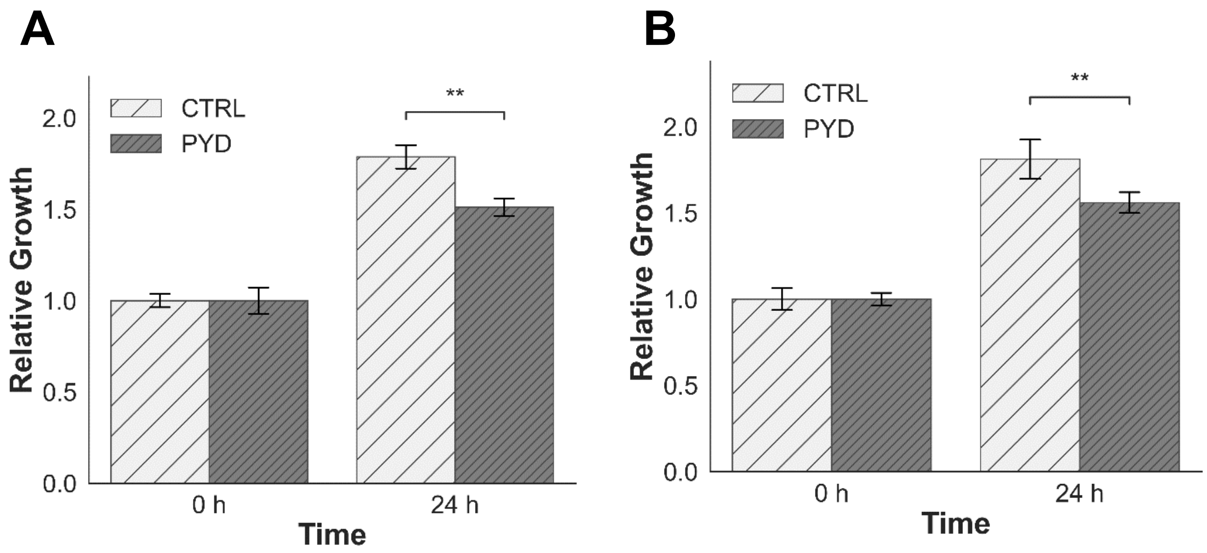

Figure S4. Relative growth of cells after a 2 Gy radiation dose at 6 MV delivered by a clinical linear accelerator. A-B) Reduction in relative growth 24 h post irradiation for HeLa and HCT-116 cells, respectively. Cells were dosed with PYD at a concentration of 500 nM.
